# Correction: Liu et al. Current Understanding of Microbiomes in Cancer Metastasis. *Cancers* 2023, *15*, 1893

**DOI:** 10.3390/cancers16111994

**Published:** 2024-05-24

**Authors:** Jiaqi Liu, Feiyang Luo, Liyan Wen, Zhanyi Zhao, Haitao Sun

**Affiliations:** Clinical Biobank Center, Microbiome Medicine Center, Department of Laboratory Medicine, Zhujiang Hospital, Southern Medical University, Guangzhou 510280, China; 3190017003@i.smu.edu.cn (J.L.); 3210101024@i.smu.edu.cn (F.L.); biobank@smu.edu.cn (L.W.); zhanyi1998@smu.edu.cn (Z.Z.)

In the original publication [1], the authors wish to revise the arrow of the epithelial–mesenchymal transition (EMT) in the schematic representation of the mechanism of suppressing cancer metastasis in Figure 1, which was overlooked in the final proofreading.

In Figure 1, in the schematic representation of the mechanism of suppressing cancer metastasis by suppressing the epithelial–mesenchymal transition (EMT), E-calmodulin should be upregulated instead of downregulated. Therefore, an upward arrow should be used instead of a downward arrow.

This is the corrected figure:

**Figure 1:**
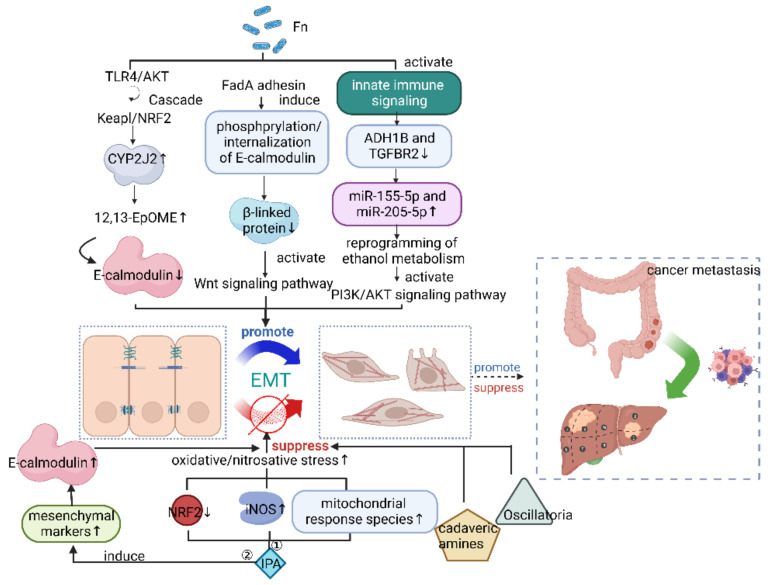
Microbiome affects cancer metastasis through epithelial–mesenchymal transition (EMT). *Fn*, *Fusobacterium nucleatum*; ADH1B, alcohol dehydrogenase 1B; TGFBR2, transforming growth factor β receptor 2; IPA, indolepropionic acid. Figure created with BioRender.com on 14 January 2023.

The authors apologize for any inconvenience caused and state that the scientific conclusions are unaffected. The original article has been updated.

## References

[1] Liu J., Luo F., Wen L., Zhao Z., Sun H. (2023). Current Understanding of Microbiomes in Cancer Metastasis. Cancers.

